# Methylene Blue Protects against TDP-43 and FUS Neuronal Toxicity in *C. elegans *and *D. rerio*

**DOI:** 10.1371/journal.pone.0042117

**Published:** 2012-07-27

**Authors:** Alexandra Vaccaro, Shunmoogum A. Patten, Sorana Ciura, Claudia Maios, Martine Therrien, Pierre Drapeau, Edor Kabashi, J. Alex Parker

**Affiliations:** 1 Université de Montréal Hospital Research Centre, Montréal, Québec, Canada; 2 Département de pathologie et biologie cellulaire and Groupe de recherche sur le système nerveux central, Université de Montréal, Montréal, Canada; 3 Centre of Excellence in Neuromics, Université de Montréal, Montréal, Canada; Baylor College of Medicine, Jiao Tong University School of Medicine, United States of America

## Abstract

The DNA/RNA-binding proteins TDP-43 and FUS are found in protein aggregates in a growing number of neurodegenerative diseases, including amyotrophic lateral sclerosis (ALS) and related dementia, but little is known about the neurotoxic mechanisms. We have generated *Caenorhabditis elegans* and zebrafish animal models expressing mutant human TDP-43 (A315T or G348C) or FUS (S57Δ or R521H) that reflect certain aspects of ALS including motor neuron degeneration, axonal deficits, and progressive paralysis. To explore the potential of our humanized transgenic *C. elegans* and zebrafish in identifying chemical suppressors of mutant TDP-43 and FUS neuronal toxicity, we tested three compounds with potential neuroprotective properties: lithium chloride, methylene blue and riluzole. We identified methylene blue as a potent suppressor of TDP-43 and FUS toxicity in both our models. Our results indicate that methylene blue can rescue toxic phenotypes associated with mutant TDP-43 and FUS including neuronal dysfunction and oxidative stress.

## Introduction

ALS is a late-onset progressive neurodegenerative disease affecting motor neurons and ultimately resulting in fatal paralysis [1], [2]. The majority of cases are sporadic but ∼10% of patients have an inherited familial form of the disease. Dominant mutations in SOD1 (copper/zinc superoxide dismutase 1) account for ∼20% of familial ALS cases and ∼1% of sporadic cases [1]. A recent biochemical approach identified cytosolic aggregates of TDP-43 in ALS and frontotemporal lobar dementia pathological tissue [3]. This breakthrough discovery was quickly followed by the identification of TDP-43 mutations in ALS patients by numerous groups [3]–[6]. TDP-43 is a multifunctional RNA/DNA binding protein and mutations in the related protein FUS have also been found in ALS patients [7] though the molecular pathology induced by mutant TDP-43 and FUS is not understood. The mislocalization and subsequent aggregation of TDP-43 has been observed in pathological tissue obtained from a number of neurological disorders including frontotemporal lobar dementia, Parkinson's disease, polyglutamine diseases and several myopathies [8]. Similarly, FUS inclusions have been observed in clinically distinct forms of frontotemporal lobar dementia and the polyglutamine diseases [8] suggesting that TDP-43 and FUS may be a common pathogenic factor in neurodegeneration. Furthermore, TDP-43 and FUS interact genetically (though not with SOD1) in zebrafish [9] and *Drosophila* [10] indicating that they may act in a common pathway. In the absence of knowledge concerning the biochemical defects caused by these ALS-related mutations in TDP-43 and FUS, the use of *in vivo* models is currently the most promising approach available to further our understanding of pathogenic mechanisms as well as for therapeutic discovery for ALS.

Indeed a number of chemical and drug screens have been published using *in vivo* models such as *C. elegans* and zebrafish [11]–[14]. These model organisms offer several advantages over mouse models for cheaper, faster and large-scale initial drug screening and target characterization. For instance, it is possible to rapidly produce large numbers of mutant offspring that can be assayed in liquid culture in multiwell plates and treated with various compounds to determine if disease phenotypes are rescued. Moreover, these organisms have relatively short reproductive cycles, they are easy to manipulate genetically, and their transparency permits visual assessment of developing cells and organs. Also, biochemical pathways are highly conserved between *C. elegans*, zebrafish and humans. We developed novel *in vivo* genetic models of mutant human TDP-43 and FUS in *C. elegans* [15] and zebrafish [9], [16], [17]. Our models exhibit several aspects of ALS including motor neuron degeneration, axonal deficits and progressive paralysis. The goal of this study was to test the ability of our *in vivo* models to identify neuroprotective compounds and determine their suitability as a platform for pre-clinical drug discovery in ALS. We focused on three compounds with known neuroprotective properties in an attempt to identify small molecules that might rescue disease phenotypes observed in our models. Here, we show that methylene blue (MB) restores normal motor phenotypes in *C. elegans* and zebrafish ALS models.

## Results

### Methylene blue rescues mutant TDP-43 and FUS behavioral phenotypes in *C. elegans*

**Figure 1:**
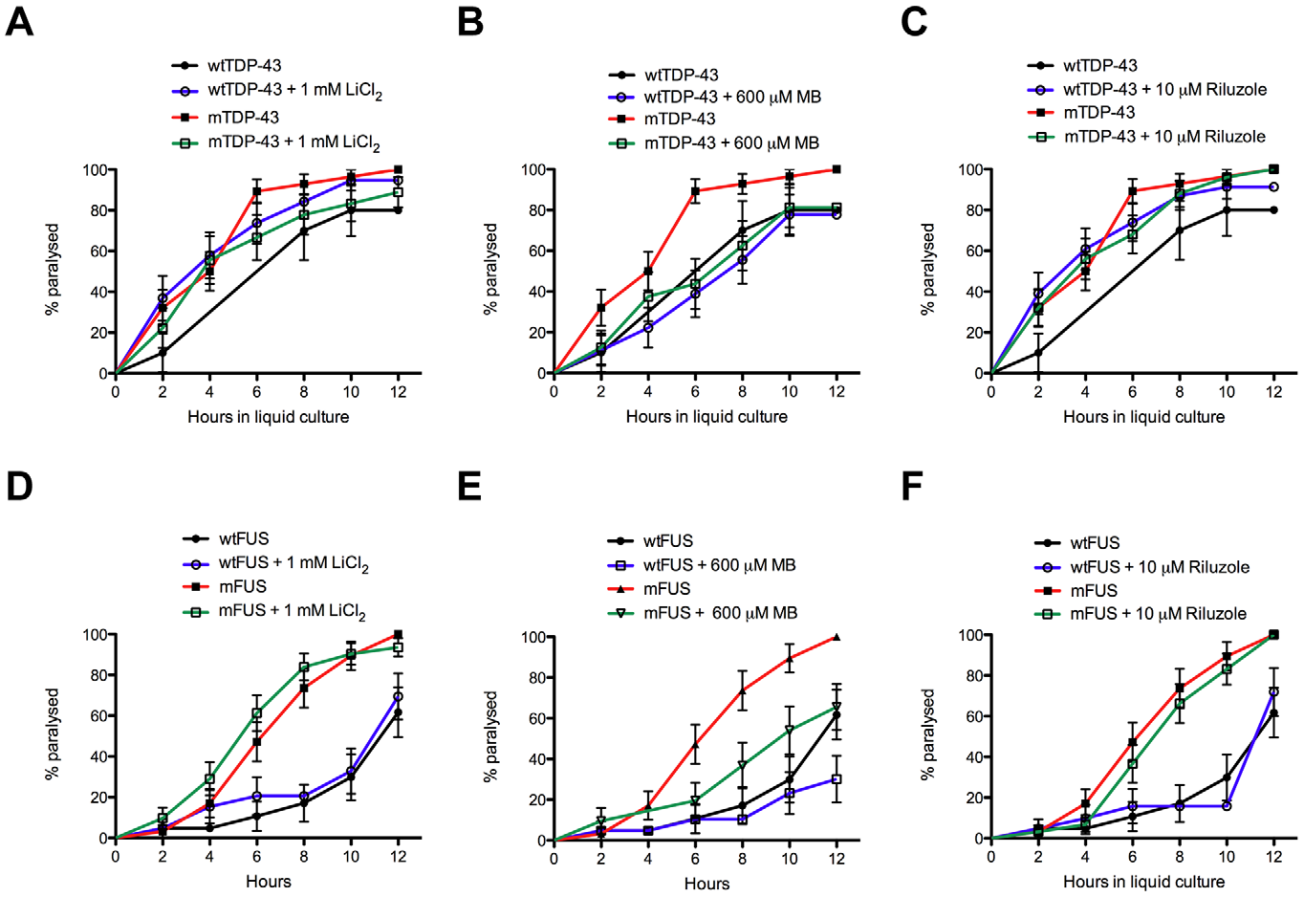
Methylene blue suppresses mTDP-43 and mFUS associated paralysis in *C. elegans*. We screened for suppression of TDP-43 (A–C) and FUS (B–D) induced paralysis in liquid culture by lithium chloride (LiCl_2_), methylene blue (MB) or riluzole. MB significantly reduced the rate of paralysis in (B) mTDP-43 and (D) mFUS transgenics compared to untreated mutant transgenic worms (P<0.05) with no effect on wild type transgenic controls.

Using *C. elegans* transgenics that express mutant TDP-43 or FUS (TDP-43[A315T] or FUS[S57Δ], referred to herein as mTDP-43 and mFUS respectively) in motor neurons [15] we evaluated the efficacy of these models as drug discovery tools by testing three compounds with known clinically neuroprotective properties: lithium chloride, MB and riluzole [18], [19]. The mTDP-43 and mFUS transgenic worms show adult-onset, progressive motility defects leading to paralysis when grown under standard laboratory conditions on solid agar plates over the course of 10 to 12 days [15]. However, worms grown in liquid culture exhibit a swimming behavior that is more vigorous than crawling on plates and accelerates neuronal dysfunction in the TDP-43 and FUS transgenics [15]. As a result, paralysis phenotypes manifest in a matter of hours instead of days. We took advantage of this phenomenon to develop a chemical screening assay to identify compounds that suppress the acute paralysis of mTDP-43 and mFUS transgenic worms grown in liquid culture. With this assay we tested if lithium chloride, MB or riluzole could suppress the paralysis caused by mTDP-43 and mFUS (**Figure 1**). Of the three compounds tested, we observed that MB reduced the rate of paralysis for mTDP-43 and mFUS transgenics with no effect on wild type TDP-43 (wtTDP-43) or wild type FUS (wtFUS) control strains (**Figures 1B, 1E**). Furthermore MB had no significant effect on movement phenotypes for wild type, non-transgenic N2 worms (**Figure S1A**).

**Figure 2:**
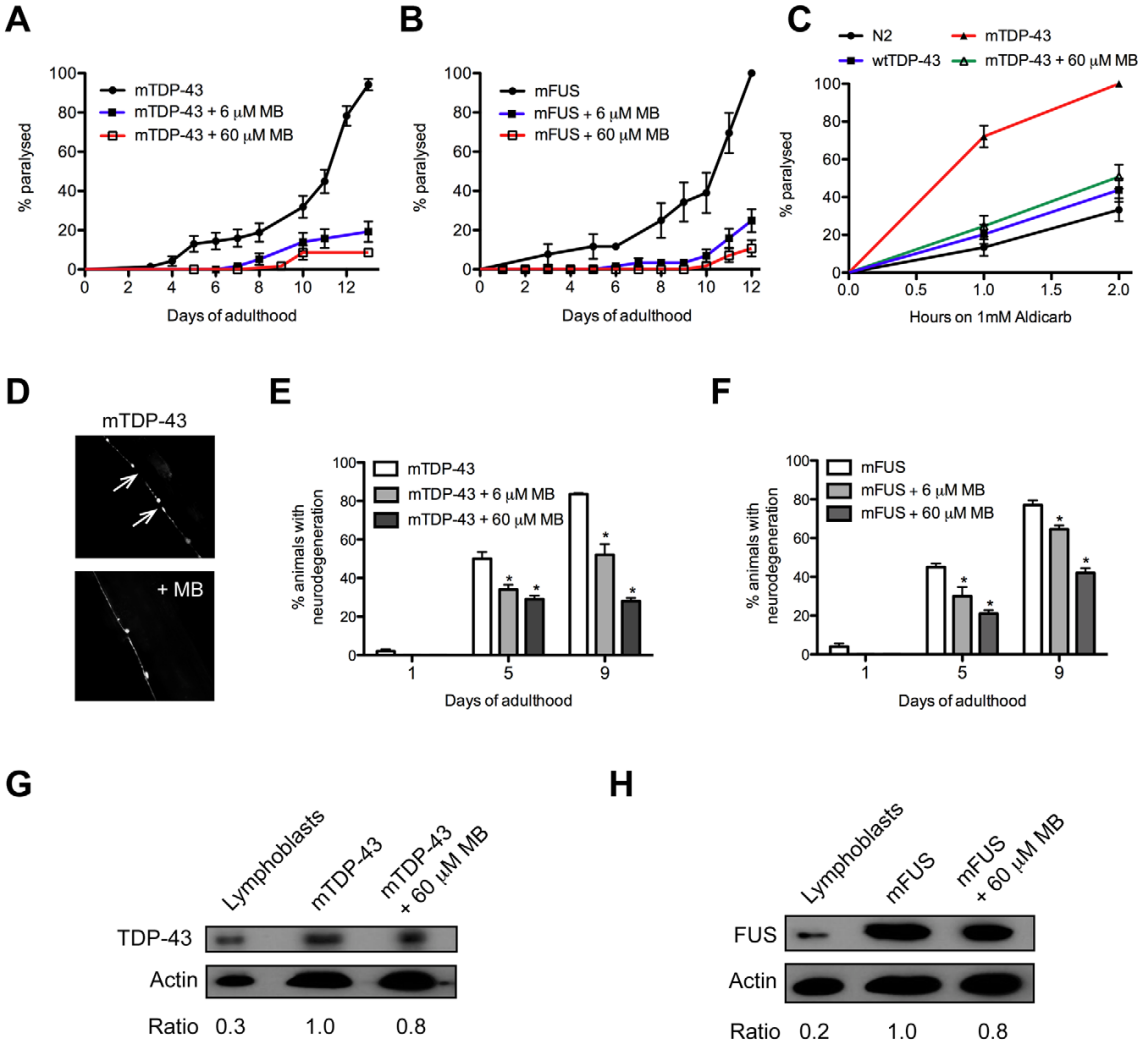
Methylene blue reduces TDP-43 and FUS neuronal toxicity. mTDP-43 and mFUS transgenics were grown on plates and assayed for various phenotypes. (**A**) MB reduced mTDP-43 induced paralysis in worms at two doses compared to untreated controls (P<0.001). (**B**) MB at two doses reduced mFUS induced paralysis in worms compared to untreated controls (P<0.001). (**C**) Aldicarb induced paralysis for mTDP-43 worms is significantly higher for mTDP-43 worms compared to non-transgenic N2 worms or transgenic wtTDP-43 controls (P<0.001). MB reduced aldicarb induced paralysis of mTDP-43 worms back to non-transgenic N2 and wtTDP-43 levels. (**D**) Representative photos of motor neuron degeneration phenotypes observed in mTDP-43 transgenic worms. Similar phenotypes were observed for mFUS transgenics. Degeneration is most frequently seen as gaps (white arrows) along neuronal processes. MB reduced the age-dependent degeneration of motor neurons in (**E**) mTDP-43 and (**F**) mFUS transgenic worms (*P<0.001 compared to untreated transgenics). MB did not affect the expression of mutant proteins in (**G**) mTDP-43 or (**H**) mFUS strains as determined by western blotting of protein extracts from transgenic worms grown with or without MB. Immunoblotting of human lymphoblasts was used as a size control.

To ensure that suppression of paralysis was not an artifact of the liquid culture assay and to confirm that MB retained its rescuing activity in the context of aging we retested it at two doses (6 and 60 µM) for mTDP-43 and mFUS worms grown on plates and observed a reduction in the rates of paralysis for treated animals compared to untreated controls (**Figures 2A, B**). The paralysis phenotype likely results from impaired synaptic transmission at the neuromuscular junction as shown by the hypersensitivity of the mTDP-43 worms to the acetylcholine esterase inhibitor aldicarb. mTDP-43 animals treated with MB showed reduced sensitivity to aldicarb, matching the response from control strains, suggesting that MB restores synaptic function in animals expressing mutant proteins (**Figure 2C**). Transgenic *C. elegans* expressing ALS-related mutations mTDP-43 or mFUS in motor neurons also show age-dependent degeneration most frequently observed as gaps or breaks along neuronal processes [15]. These neurodegenerative phenotypes were significantly reduced by treatment with MB (**Figures 2D, E, F**) and did not change mTDP-43 or mFUS transgene expression (**Figures 2G, H**).

### Methylene blue rescues motor phenotypes in mutant TDP-43 and FUS zebrafish

**Figure 3:**
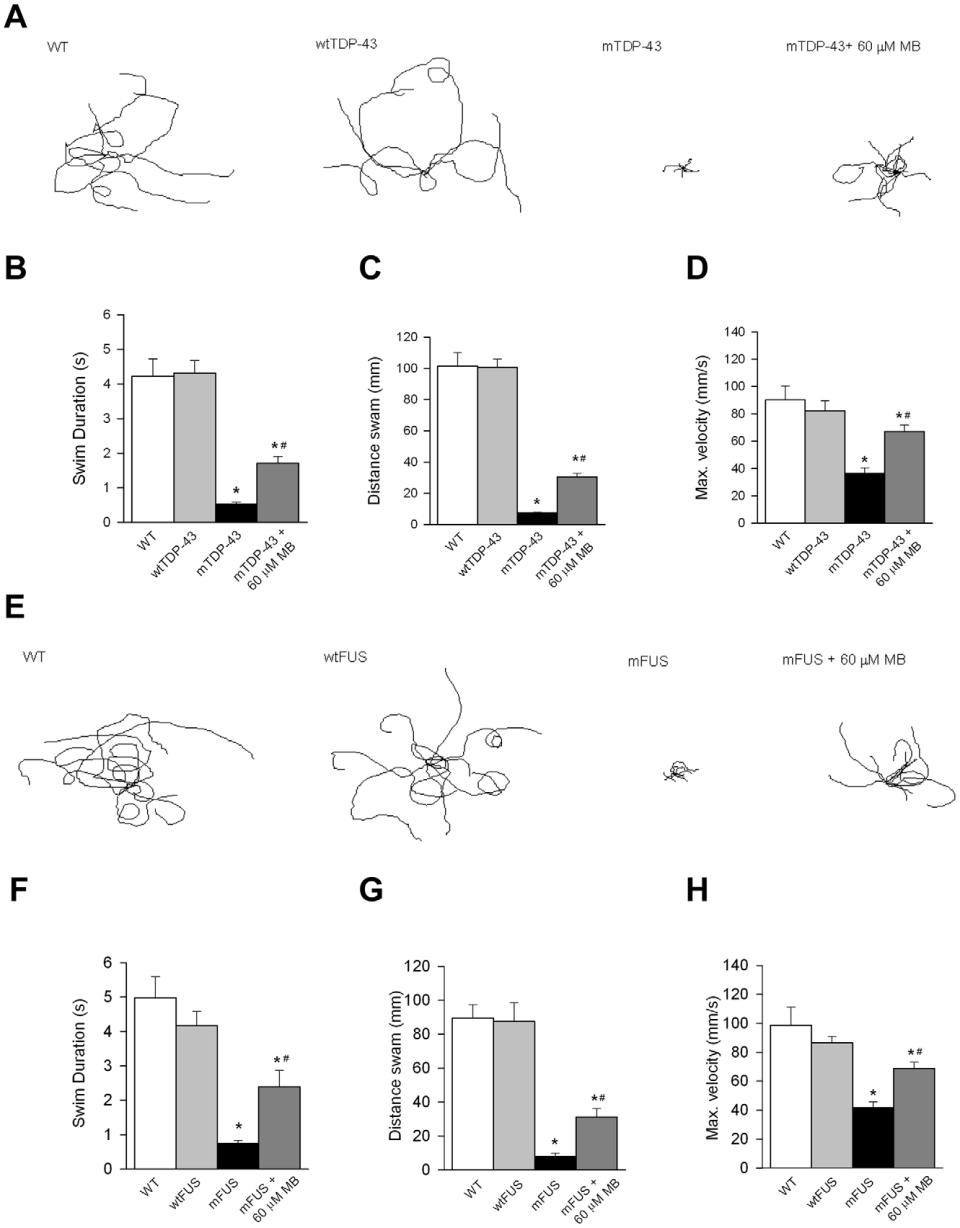
Methylene blue reduces motor deficits in zebrafish expressing mutant TDP-43 or FUS. (**A**) Representative traces of TEER phenotypes in wild type (WT), wtTDP-43, mTDP-43 and mTDP-43+MB. MB improved the swim duration (**B**), distance swam (**C**) and maximum swimming velocity (**D**) of mTDP-43 fish. (**E**) Representative traces of TEER phenotypes in WT, wtFUS, mFUS and mFUS+MB. Application of MB led to a significant improvement in the swim duration (**F**), distance swam (**G**) and maximum swimming velocity (H) of mTDP-43 fish. * denotes significant difference from WT, P<0.001; # significantly different from mutant fish P<0.05.

**Figure 4:**
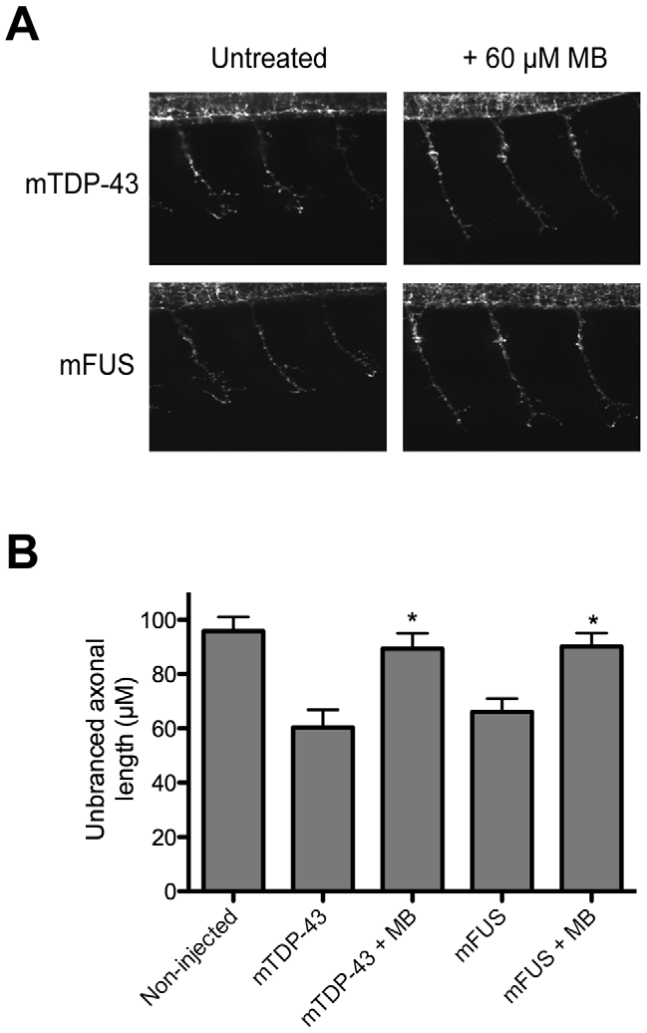
Methylene blue reduces axon defects in zebrafish expressing mutant TDP-43 or FUS. (**A**) Appearance of motor neurons in mTDP-43 and mFUS transgenic zebrafish with and without MB treatment. (**B**) MB reduced the unbranched axon length phenotype of motor neurons in mTDP-43 and mFUS transgenic zebrafish (*P<0.01 compared to untreated transgenics).

To test if MB had protective effects beyond *C. elegans* we turned to zebrafish. First, as in worms, we observed that MB had no effect on the movement phenotypes of wild type non-transgenic fish (**Figure S1B, C, D, E**). Zebrafish expressing mTDP-43[G348C] or mFUS[R521H] have impaired swimming as assessed by their ability to produce a touch-evoked escape response (TEER) [9], [16]. mTDP-43 fish showed a greatly reduced TEER compared to non-transgenic or wtTDP-43 fish (**Figure 3A**). mTDP-43 fish treated with 60 µM MB showed improved swimming response including swim duration, distance swam and maximum swim velocity (**Figures 3A, B, C, D**). Zebrafish expressing mFUS also show greatly reduced swimming activity compared to wild type or wtFUS fish and the swimming phenotype of mFUS fish was greatly improved when treated with 60 µM MB (**Figures 3E, F, G, H**). Besides behavioral defects, immunohistochemical analyses show that transgenic zebrafish expressing mTDP-43 or mFUS also displayed abnormally shortened and branched motor neuron axonal processes as observed by the unbranched axonal length (UAL) quantification [9], [16] and this phenotype was rescued by incubation with either 30 or 60 µM MB (**Figures 4A, B**). These results demonstrate that MB can significantly reduce the motor neuron phenotypes elicited by expression of mTDP-43 and mFUS both in *C. elegans* and zebrafish genetic models of disease.

### Methylene blue protects against oxidative stress in *C. elegans* and zebrafish

**Figure 5:**
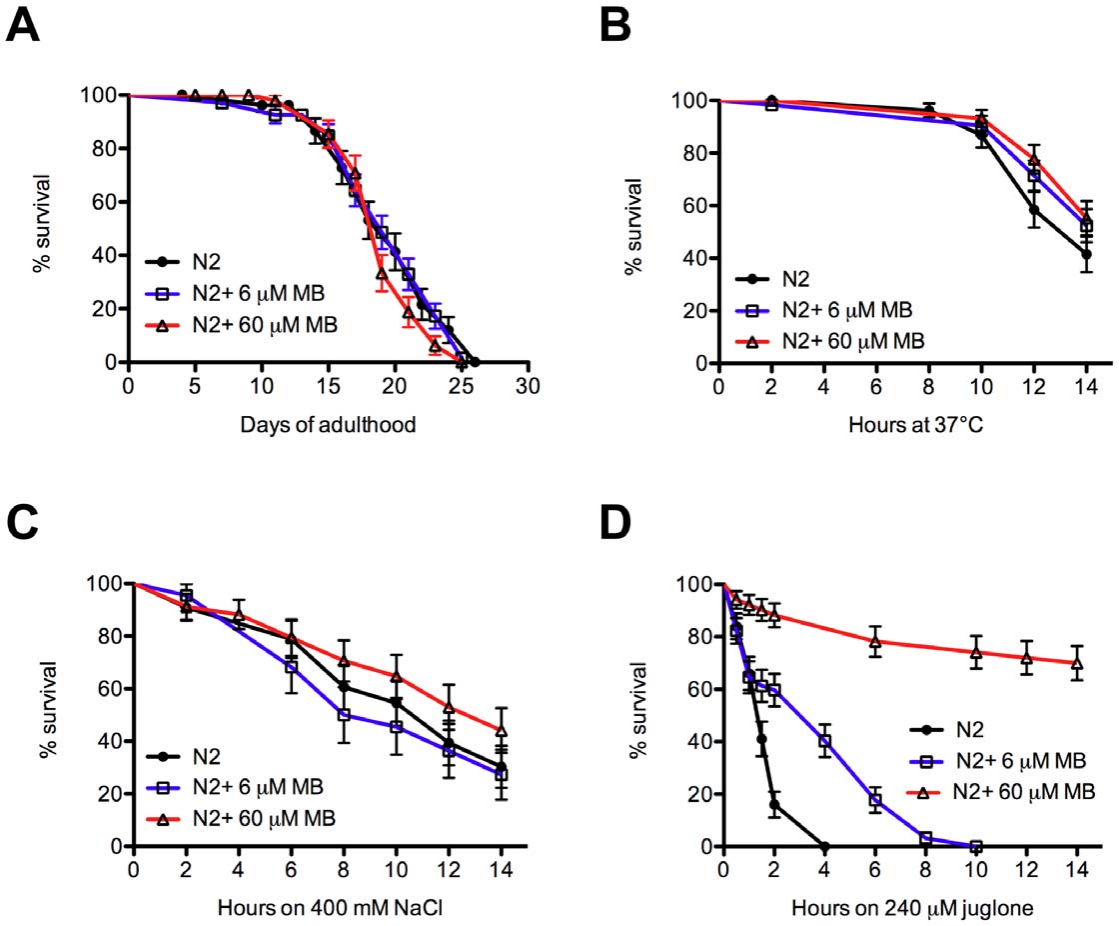
Methylene blue protects against oxidative stress in *C. elegans*. (**A**) N2 worms grown on plates with MB had lifespans indistinguishable from untreated worms (see also Table S1). (**B**) Worms grown on MB and subjected to thermal stress showed similar survival rates compared to untreated N2 worms. (**C**) N2 worms treated with MB showed similar rates of survival compared to untreated worms when subjected to hyperosmolarity. (**D**) MB had a dose-dependent protective effect on N2 worms against oxidative stress and mortality when grown on plates containing juglone (P<0.001 for MB treated N2 worms compared to untreated worms).

Since we observed that MB rescued paralysis in transgenic models of mTDP-43 or mFUS, we sought to further examine the protective effects of MB in an aging and stress context. First, MB treatment had no effect on the lifespan of wild type N2 worms suggesting that its cellular protection mechanisms are not due to non-specific effects from extended longevity (**Figure 5A** **, Table S1**). To test for protective effects against environmental stress we tested wild type N2 worms for their ability to withstand lethal exposure to thermal, hyperosmotic or oxidative stresses. We observed that MB offered no protection to worms subjected to elevated temperature or hyperosmotic stress from treatment with NaCl as their survival rate was indistinguishable from untreated control animals (**Figures 5B, C**). Juglone is a natural aromatic compound found in the black walnut tree that induces high levels of oxidative stress within cells [20]. Juglone is highly toxic to wild type N2 worms and causes complete mortality after approximately 4 hours in our assay. We observed that MB provided significant protection against oxidative stress since wild type N2 worms were resistant to juglone in a dose dependent manner (**Figure 5D**). These data suggest that MB is specific in its cell protection capabilities and helps overcome oxidative stress conditions in *C. elegans*.

**Figure 6:**
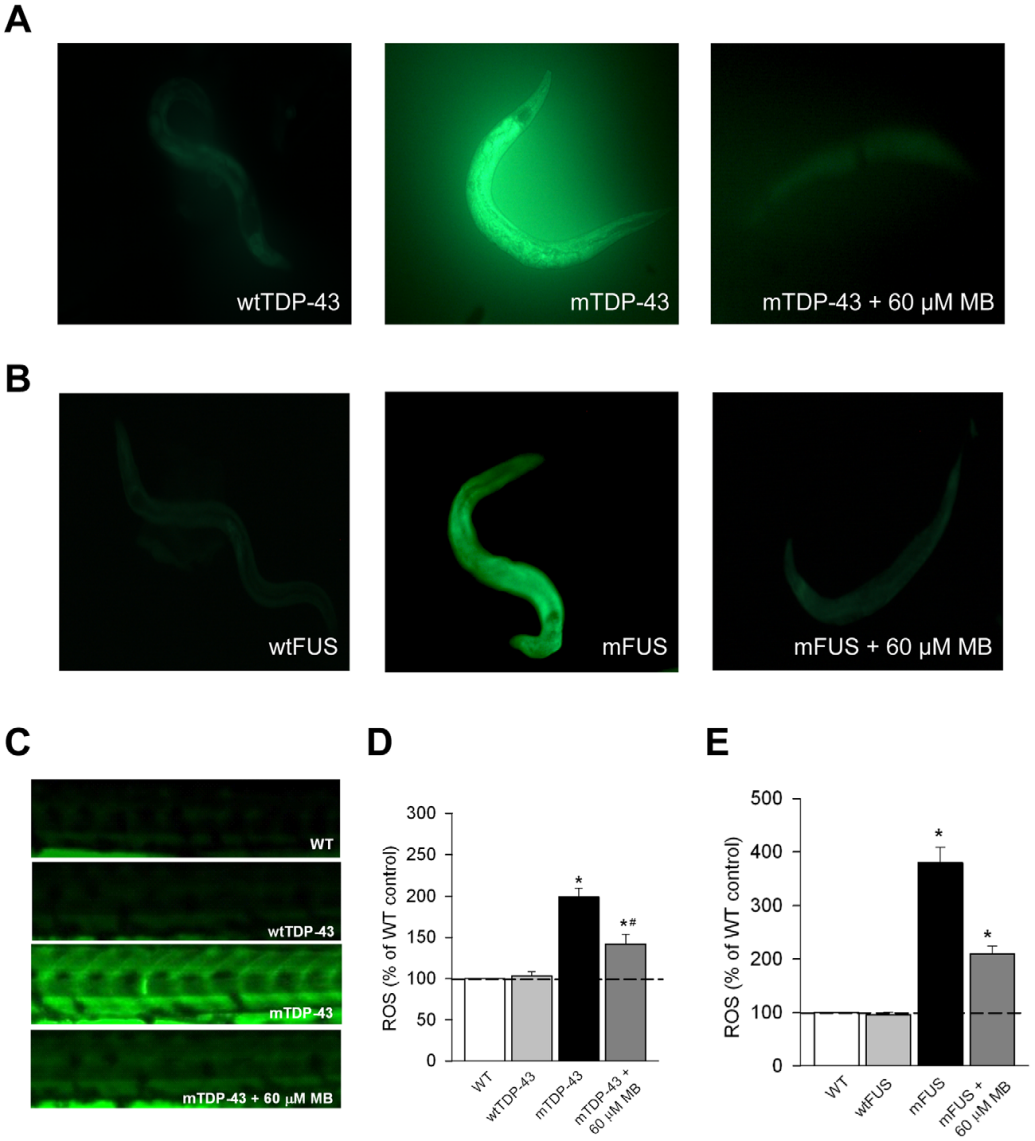
Methylene blue reduces oxidative stress in *C. elegans* and zebrafish transgenics. Oxidative stress was measured in transgenic worms and zebrafish with the dihydrofluorescein diacetate (DHF) that fluoresces when exposed to intracellular peroxide. (A) mTDP-43 worms, but not wtTDP-43 transgenics have a higher level of oxidative stress when stained with DHF. mTDP-43 worms treated with MB and then stained with DHF show a remarkable reduction in fluorescence. (B) wtFUS worms show no fluorescence when stained with DHF compared to mFUS worms. mFUS worms treated with MB and then stained with DHF showed reduced fluorescence. (**C**) Wild type (WT) zebrafish and zebrafish expressing wtTDP-43 show very low levels of fluorescence when stained with DHF compared to mTDP-43 fish. Treatment with MB reduced fluorescence in DHF stained fish. (**D**) Quantification of fluorescence of DHF stained fish shows that MB treatment significantly reduced fluorescence in mTDP-43 fish (*P<0.001, *#P<0.01). (**E**) MB significantly reduced fluorescence in DHF stained mFUS zebrafish (*P<0.001).

Since we showed that MB confers protection to wild type N2 worms under oxidative stress in a dose dependent manner we hypothesized that MB may help reduce oxidative damage in mTDP-43 worms. To test this hypothesis we stained our TDP-43 transgenic strain with dihydrofluorescein diacetate (DHF), a compound known to fluoresce when exposed to intracellular peroxides associated with oxidative stress [21]. We observed no DHF signal from wtTDP-43 transgenics but strong fluorescence from mTDP-43 worms (**Figure 6A**). The fluorescence observed in the mTDP-43 transgenics was reduced when treated with 60 µM MB (**Figure 6A**). We observed a similar effect with our FUS transgenics, with no DHF signal from wtFUS animals, but strong fluorescence from mFUS worms that was reduced upon MB treatment (**Figure 6B**). Extending our findings we examined oxidative stress with DHF in mTDP-43 and mFUS fish. Similar to worms, we observed a strong fluorescent signal in mTDP-43 fish compared to non-transgenic or wtTDP-43 fish and that this signal was reduced by treatment with MB (**Figures 6C, D**). MB also reduced the fluorescent signal in mFUS fish stained with DHF (**Figure 6E**). These data suggest that MB reduces the general level of oxidative status generated by the expression of mutant proteins *in vivo*.

### Reduced neuroprotection from late administration of methylene blue

**Figure 7:**
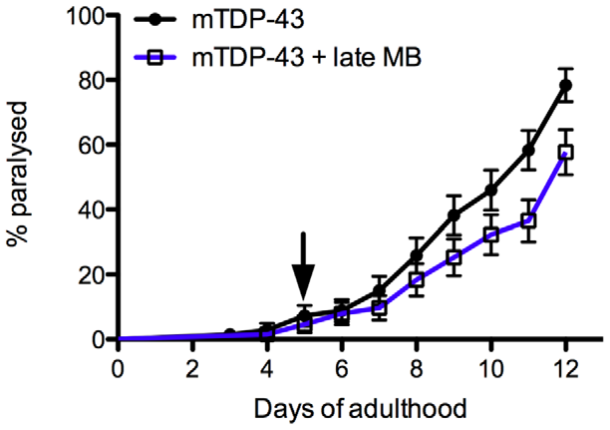
Diminished neuroprotection from late administration of methylene blue. mTDP-43 worms grown on normal plates and switched to plates supplemented with 60 µM MB at day 5 (indicated by the arrow) of adulthood (late MB) showed a modest but significant reduction in paralysis compared to untreated worms (P<0.05).

In the previous experiments worms and fish were treated with MB from hatching. We tested whether the timing of treatment had an effect on the magnitude of neuroprotection by growing mTDP-43 worms on normal plates and transferring them at day 5 of adulthood to plates supplemented with MB. We observed that late administration of MB reduced paralysis with approximately 55% of treated animals becoming paralysed at day 12 of adulthood compared to a paralysis rate of approximately 80% for untreated animals (**Figure 7**). However the extent of rescue by late MB administration was far less than the approximate 10% paralysis rate observed for mTDP-43 animals grown on MB plates from hatching (**Figure 2A**). These data suggest that early administration of MB is more effective at reducing mTDP-43 toxicity than intervention in older animals.

## Discussion

In this study we demonstrated that our *C. elegans* and zebrafish ALS models can be used to identify neuroprotective molecules which represents the first *in vivo* chemical genetic screening platform for ALS. With this platform we discovered that MB is a potent suppressor of mTDP-43 and mFUS motor neuron toxicity *in vivo*. In both worms and fish MB corrected motor deficits and reduced the level of oxidative stress associated with the expression of mutant proteins.

MB is a pleiotropic molecule with a long and varied history of medical use [18] but in the context of neurodegeneration MB has been reported to prevent amyloid-β and tau aggregation *in vitro* [22], [23]. A previous study also showed that the treatment of cells with MB inhibited the formation of TDP-43 aggregates [24] suggesting this compound might be appropriate for the treatment of ALS and other dementias. The efficacy of MB as a neuroprotective compound has been examined in Alzheimer's disease and ALS models where in some studies it is protective while in others it has no effect [24]–[28]. We decided to include this compound in our assay from which we identified MB as a potent suppressor of mTDP-43 and mFUS toxicity in both *C. elegans* and zebrafish. However, our data do not agree with a recent study examining the effects of MB in a TDP-43 mouse model [26]. Mutant TDP-43[G348C] mice treated with MB showed no improvement in motor phenotypes as determined by the rotarod assay. Furthermore no difference in the cytoplasmic localization of TDP-43 was observed in treated mice.

Worms and fish live in aqueous media and a simple explanation for their greater susceptibility may be that they are more permeable to MB. We further hypothesize that the differences in MB efficacy might also be due to variations in timing for delivery of the compound. Specifically, our worms and fish were treated with MB from hatching whereas the TDP-43 mice were treated at 6 months. To confirm this hypothesis we treated mTDP-43 worms with MB at day 5 of adulthood and observed that late administration of the compound was significantly less effective at reducing paralysis. Thus, perhaps earlier (pre-clinical) treatment with MB may have greater effects in mouse models for ALS. Additionally there may be differences between the models since our worm and fish models capture a clinical aspect of ALS, namely progressive paralysis in animals expressing mTDP-43 that is absent from the TDP-43 mouse model.

Aging is a risk factor common to a number of neurodegenerative disorders including ALS, and oxidative stress is suspected to play a key role in the development of the disease by contributing to aging [29], [30]. Indeed, interactions between genetic, environmental, and age-dependent risk factors have been hypothesized to trigger disease onset [31]. Consequently, we investigated the impact of MB treatment focusing on aging and stress response. Our *C. elegans* data are in agreement with the survival data from the mouse studies where we observed no effect on lifespan in MB treated worms even though there was a positive effect on multiple phenotypes associated with mTDP-43 or mFUS. Thus, at least in simple systems lifespan effects can be uncoupled from neuroprotection but it remains to be seen if the same is true for mouse models of neurodegeneration.

In our previous work we showed that our TDP-43 and FUS transgenic *C. elegans* models exhibited no difference in lifespan compared to non-transgenic worms [15]. Thus, the paralysis phenotypes observed in our models specifically reflect the consequences of the expression of TDP-43 and FUS in motor neurons and are not due to secondary effects from general sickness and reduced lifespan. Therefore, it may be difficult to detect significant improvement on motor function or reflex phenotypes after MB treatment in mice showing generalized defects instead of treating problems resulting from TDP-43 or FUS proteotoxicity alone.

Finally, the TDP-43 mouse study did not examine the effects of MB on synaptic function or oxidative stress where we see clear effects in the worm and zebrafish models. MB can interact with nitric oxide synthase and also has an antioxidant potential by decreasing the generation of reactive oxygen species [32]. Using *C. elegans* we showed that MB specifically decreased the sensitivity of wild type worms to oxidative stress. We also investigated the impact of MB treatment in the formation of reactive oxygen species in both *C. elegans* and *D. rerio* and have observed a significant reduction in the generation of reactive oxygen species. Consistent with the literature [33], our data suggest that MB counteracts oxidative stress to provide protection against proteotoxicity in both our *in vivo* models. Synaptic function was also restored after treatment with MB in transgenic mTDP-43 worms suggesting that this compound might also have an effect on synaptic transmission.

In summary, we present novel *in vivo* chemical genetic screening assays that may be useful for ALS drug discovery. Using two genetic models for ALS we report here that MB acts through reduction of oxidative stress and also restoration of normal synaptic function in genetic models of ALS. In addition, an important issue here is that in simple systems like *C. elegans*, lifespan effects can be uncoupled from neuroprotection. The next step will be to unravel MB's exact target and mechanism of action to develop compounds with more specific activities and also to capitalize on the strength of our assays to screen additional compounds as potential therapeutics in ALS.

## Materials and Methods

### *C. elegans* experiments

#### *C. elegans* strains

Strains used in this study include: N2, *gas-1(fc21), let-60(ga89), oxIs12[unc-47p::GFP;lin-15(+)], xqIs98[unc-47::FUS[S57Δ];unc-119(+)], xqIs132[unc-47::TDP-43-WT;unc-119(+)], xqIs133[unc-47::TDP-43[A315T];unc-119(+)]* and *xqIs173[unc-47::FUS-WT;unc-119(+)]*.

#### *C. elegans* liquid culture assay

Young adult TDP-43 or FUS transgenic worms were distributed in 96-wells plate (20 µl per well; 20–30 worms per well), containing DMSO or test compounds and incubated for up to 12 hours at 20°C on a shaker. Compounds and final concentrations tested were 1 mM lithium chloride, 600 µM methylene blue, and 10 µM riluzole. The motility test was assessed by microscopy every 2 hours. Compounds were purchased from Sigma-Aldrich (St-Louis, MO).

#### *C. elegans* drug testing on plates

Worms were grown on standard NGM plates with or without compounds. For worms expressing mFUS or mTDP-43, animals were counted as paralyzed if they failed to move upon prodding with a worm pick. Worms were scored as dead if they were immotile, showed no pharyngeal pumping and failed to move their head after being prodded in the nose. The final concentrations of methylene blue tested in plates either 6 or 60 µM.

#### Fluorescence microscopy

For scoring axons from transgenic mFUS and mTDP-43 worms, synchronized animals were selected at days 1, 5 and 9 of adulthood for visualization of motor neurons *in vivo* with the *unc-47p*::GFP transgenic reporter. Animals were immobilized in M9 with 5 mM levamisole and mounted on slides with 2% agarose pads. Motor neurons were visualized with a Leica CTR 6000 and a Leica DFC 480 camera. A minimum of 100 animals was scored per treatment over 4–6 trials. Animals showing gaps or breaks along motor neuron processes were scored as positive for the degeneration phenotype. The mean and SEM were calculated for each trial and two-tailed *t*-tests were used for statistical analysis. For visualization of fluorescence after treatment with dihydrofluorescein diacetate, L4 animals were grown on NGM plates or NGM plates with methylene blue and examined for fluorescence with the Leica system described above.

#### Lifespan assays

Worms were grown on NGM or NGM+60 µM methylene blue and transferred on NGM-FUDR or NGM-FUDR+60 µM methylene blue. 20 animals/plate by triplicates were tested at 20°C from adult day 1 until death. Worms were declared dead if they were immotile and did not respond to tactile or heat stimulus.

#### Stress assays

For oxidative stress tests, worms were grown on NGM or NGM+60 µM methylene blue and transferred to NGM plates +240 µM juglone at adult day 1. For thermal resistance worms were grown on NGM or NGM 60 µM methylene blue and put at 37°C at adult day 1. For osmotic resistance worms were grown on NGM or NGM+60 µM methylene blue and put on 400 mM NaCl plates at adult day 1. For all assays, worms were evaluated for survival every 30 min for the first 2 hours and every 2 hours after up to 14 hours. Nematodes were scored as dead if they were immotile and unable to move in response to heat or tactile stimuli. For all tests worms, 20 animals/plate by triplicates were scored.

#### Dihydrofluorescein diacetate assay

For visualization of oxidative damage in the transgenic strains the worms were incubated on a slide for 30 min with 5 µM dihydrofluorescein diacetate dye and then washed with 1× PBS three times. After the slide was fixed, fluorescence was observed with the Leica system described above.

#### Worm lysates

Worms were collected in M9 buffer, washed 3 times with M9 and pellets were placed at −80°C overnight. Pellets were lysed in RIPA buffer (150 mM NaCl, 50 mM Tris pH 7.4, 1% Triton X-100, 0.1% SDS, 1% sodium deoxycholate)+protease inhibitors (10 mg/ml leupeptin, 10 mg/ml pepstatin A, 10 mg/ml chymostatin LPC;1/1000). Pellets were passed through a 27_1/2_ G syringe 10 times, sonicated and centrifuged at 16,000× *g*. Supernatants were collected.

#### Immunoblot

Worm RIPA samples (175 µg/well), lymphoblast cell RIPA samples (15 µg/well) were resuspended directly in 1× Laemmli sample buffer, migrated in 12.5% polyacrylamide gels, transferred to nitrocellulose membranes (BioRad) and immunoblotted. Antibodies used: rabbit anti-TDP-43 (1∶200; Proteintech), rabbit anti-FUS/TLS (1∶200; AbCam), and mouse anti-actin (1∶10000 for worms, MP Biomedicals). Blots were visualized with peroxidase-conjugated secondary antibodies and ECL Western Blotting Substrate (Thermo Scientific).

#### Statistical analysis

For paralysis and stress-resistance tests, survival curves were generated and compared using the Log-rank (Mantel-Cox) test, and a 60–100 animals were tested per genotype and repeated at least three times.

### Zebrafish experiments

#### Zebrafish maintenance

Zebrafish (*Danio rerio*) embryos were raised at 28.5°C, and collected and staged using standard methods [34]. The Comité de Déontologie de l'Expérimentation sur les Animaux (CDEA), the local animal care committee at the Université de Montréal, having received the protocol relevant to this project relating to animal care and treatment, certified that the care and treatment of animals was in accordance with the guidelines and principles of the Canadian Council on Animal Care. Further, all matters arising from this proposal that related to animal care and treatment, and all experimental procedures proposed for use with animals were reviewed and approved by the CDEA before they were initiated or undertaken. This review process was ongoing on a regular basis during the entire period that the research was being undertaken. Zebrafish embryos (no adults were used) are insentient to pain. Fish embryos were incubated overnight in each compound and examined the next day and then disposed. Zebrafish embryos were used over a two-day period then terminated.

#### In-vitro mRNA synthesis and embryo microinjection

Human FUS wild type and mutant [R521H], human TDP-43 wild type and mutant [G348C] mRNAs were transcribed from NotI-linearized pCS2+ using SP6 polymerase with the mMESSAGE Machine Kit (Ambion). This was followed by a phenol:chloroform extraction and isopropanol precipitation, and diluted in nuclease-free water (Ambion). The mRNAs were diluted in nuclease free water (Ambion) with 0.05% Fast Green vital dye (Sigma-Aldrich) at a concentration of 60 ng/µl (FUS), 25 ng/µl (TDP-43) and were pulse-injected into 1–2 cell stage embryos using a Picospritzer III pressure ejector.

#### Chemical treatments

Transient transgenics for TDP-43 [G348C] and FUS [R521H] embryos at 24 hpf were placed in individual wells in a 24 well plate and were treated overnight with methylene blue diluted in Evans solution (in mM): 134 NaCl, 2.9 KCl, 2.1 CaCl_2_, 1.2 MgCl_2_, 10 HEPES, 10 glucose, pH 7.8, 290 mOsm, with 0.1% DMSO. Behavioural touch responses were then assessed at 52–56 hpf as described in the following section.

#### Touch-evoked escape response

Zebrafish larvae were touched lightly at the level of the tail with a pair of blunt forceps and their locomotor behavior was recorded with a Grasshopper 2 Camera (Point Grey Research) at 30 Hz. The movies were then analyzed using the manual tracking plugin of ImageJ 1.45r software (NIH) and the swim duration, swim distance and maximum swim velocity of the fish were calculated.

#### Unbranched axonal length measurements

For immunohistochemical analysis of axonal projections of motor neurons, monoclonal antibody anti-SV2 (Developmental Studies Hybridoma) were used to assess the motor neuron morphology at 48 and 72 hpf. Fluorescent images of fixed embryos were taken using a Quorum Technologies spinning-disk confocal microscope mounted on an upright Olympus BX61W1 fluorescence microscope equipped with an Hamamatsu ORCA-ER camera. Image acquisition was performed with Volocity software (PerkinElmer). As previously described [16], axonal projections from primary and secondary motor neurons at a defined location in the inter somitic segments were determined. Analysis of Z-stacks by confocal microscopy was performed in three to four axonal projections per animal. The axonal length to the first branching (UAL) was determined by tracing the labeled axon from the spinal cord to the point where it branches using ImageJ (NIH). These values were averaged for each of the animal analyzed (10–30 zebrafish per condition) for the various conditions in our study.

#### Reactive Oxygen Species measurements

For *in vivo* detection of reactive oxygen species, live 2 day old embryos (2 dpf) were incubated in 5 µM 2′,7′-dichlorofluorescein diacetate (Sigma-Aldrich) for 20 minutes at 28.5°C and washed three times for 5 min with embryo media. Fluorescence was observed under a 488 nm wavelength excitation. The generation of reactive oxygen species in the larvae exposed to the chemicals was also quantitatively assessed as described elsewhere [35]. Briefly, 15 embryos were washed with cold Phosphate Buffer solution (PBS; pH 7.4) twice and then homogenized in cold buffer (0.32 mM of sucrose, 20 mM of HEPES, 1 mM of MgCl_2_, and 0.5 mM of phenylmethyl sulfonylfluoride (PMSF), pH 7.4). The homogenate was centrifuged at 15,000× g at 4°C for 20 min, and the supernatant was transferred to new tubes for further experimentation. Twenty microliters of the homogenate was added to a 96-well plate and incubated at room temperature for 5 min, after which 100 µl of PBS (pH 7.4) and 8.3 µl of DFH stock solution (10 mg/ml) were added to each well. The plate was incubated at 37°C for 30 minutes. The fluorescence intensity was measured using a microplate reader (SpectraMax M2, Molecular Device, Union City, CA, USA) with excitation and emission at 485 and 530 nm, respectively. The reactive oxygen species concentration was expressed as arbitrary emission units per mg protein.

#### Statistical analysis

All data values are given as means ± SEM. Significance was determined using one-way ANOVAs and Fisher LSD tests for normally distributed and equal variance data, Kruskal–Wallis ANOVA and Dunn's method of comparison were used for non-normal distributions.

## Supporting Information

Figure S1 **Methylene blue has no effect on wild type motility phenotypes in worms or zebrafish.** (**A**) MB had no significant effect on the motility phenotype of wild type (WT) non-transgenic N2 worms. (**B**) Representative traces of TEER phenotypes in WT zebrafish with and without MB treatment. MB did not affect the swim duration (**C**), distance swam (**D**) or maximum swimming velocity (**E**) of WT zebrafish.
(TIF)

Table S1 **Lifespan analysis for all experiments.** Related to Figure 5A. Animals that died prematurely (ruptured, internal hatching) or were lost (crawled off the plate) were censored at the time of scoring. All control and experimental animals were scored and transferred to new plates at the same time. ns: not significant.
(PDF)
