# Supplementary figures and images for: Methylene Blue Protects against TDP-43 and FUS Neuronal Toxicity in C. elegans and D. rerio
Source: PLoS One. 2012 Jul 27;7(7):e42117. doi: 10.1371/journal.pone.0042117 (PMC3407135; doi:10.1371/journal.pone.0042117)

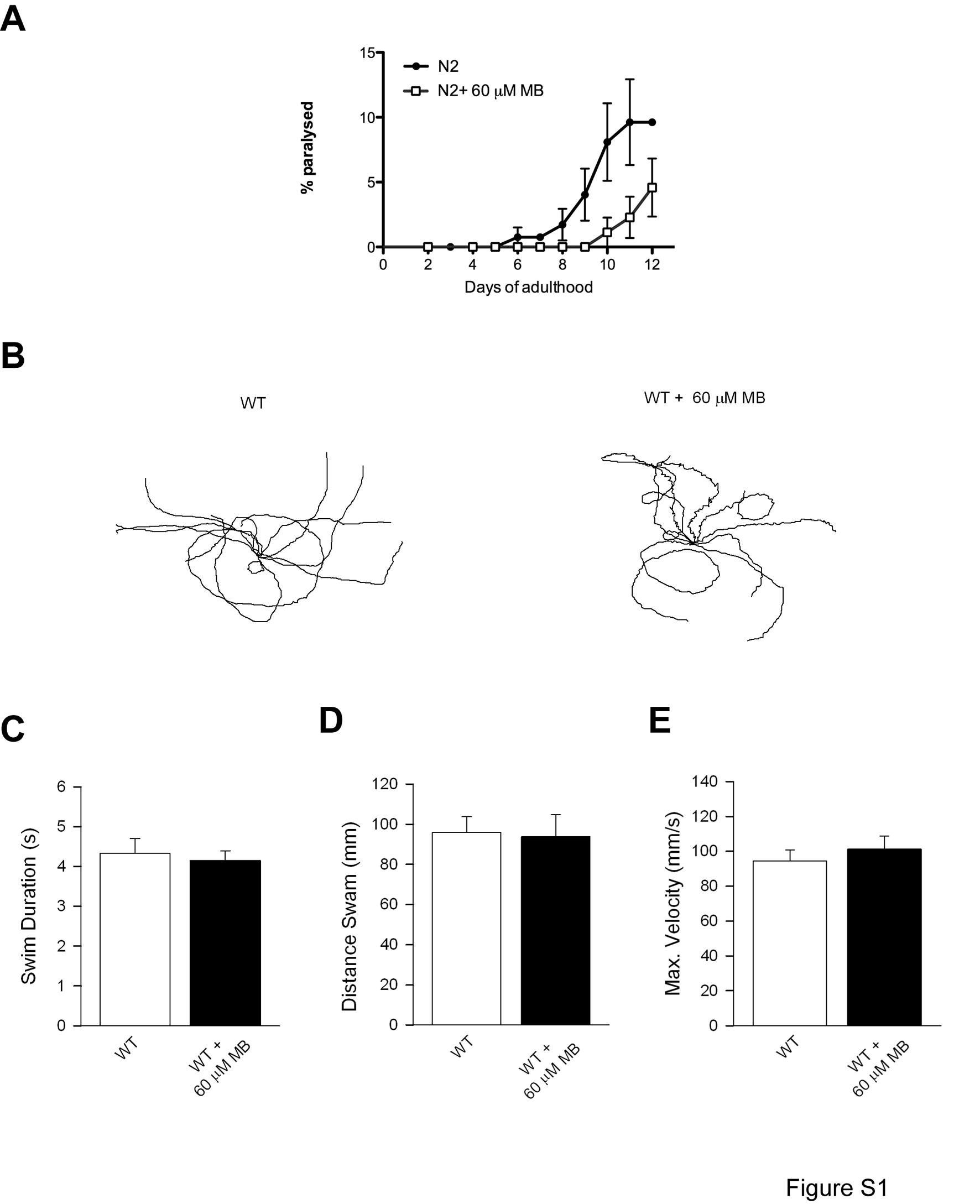

Supplement: Figure S1 — Methylene blue has no effect on wild type motility phenotypes in worms or zebrafish. (A) MB had no significant effect on the motility phenotype of wild type (WT) non-transgenic N2 worms. (B) Representative traces of TEER phenotypes in WT zebrafish with and without MB treatment. MB did not affect the swim duration (C), distance swam (D) or maximum swimming velocity (E) of WT zebrafish. (TIF) [file pone.0042117.s001.tif]
