# Supplementary material for: Methylene Blue Protects against TDP-43 and FUS Neuronal Toxicity in C. elegans and D. rerio
Source: PLoS One. 2012 Jul 27;7(7):e42117. doi: 10.1371/journal.pone.0042117 (PMC3407135; doi:10.1371/journal.pone.0042117)
Supplement: Table S1 — Lifespan analysis for all experiments. Related to Figure 5A. Animals that died prematurely (ruptured, internal hatching) or were lost (crawled off the plate) were censored at the time of scoring. All control and experimental animals were scored and transferred to new plates at the same time. ns: not significant. (PDF) [file pone.0042117.s002.pdf]

	Strains	Mean Life Span	p Value	75th Percentile (Days)	Maximum Lifespan	Total Number of Animals Died/Total
Figure 5	N2	18		22	26	59/66
	N2 + 6 μM MB	19	n.s. 0.9288	22	25	63/70
	N2 + 60 μM MB	18	n.s. 0.1773	20	25	58/64

Table S1 Lifespan analysis for all experiments. Related to Figure 5A.

Animals that died prematurely (ruptured, internal hatching) or were lost (crawled off the plate) were censored at the time of scoring. All control and experimental animals were scored and transferred to new plates at the same time. ns: not significant.
